# Generation and Performance of R132H Mutant IDH1 Rabbit Monoclonal Antibody

**DOI:** 10.3390/antib6040022

**Published:** 2017-12-01

**Authors:** Juliet Rashidian, Raul Copaciu, Qin Su, Brett Merritt, Claire Johnson, Aril Yahyabeik, Ella French, Kelsea Cummings

**Affiliations:** MilliporeSigma, 6600 Sierra College Blvd, Rocklin, CA 95677, USA; jrashidian@sial.com (J.R.); raul.copaciu@sial.com (R.C.); qin.su@sial.com (Q.S.); bmerritt@sial.com (B.M.); claire.johnson@sial.com (C.J.); ayahyabeik@sial.com (A.Y.); ella.french@sial.com (E.F.)

**Keywords:** IDH1, R132H, novel rabbit monoclonal antibody, B-cell cloning, immunohistochemistry

## Abstract

Isocitrate dehydrogenase 1 (IDH1) gene mutations have been observed in a majority of diffuse astrocytomas, oligodendrogliomas, and secondary glioblastomas, and the mutant IDH1 R132H is detectable in most of these lesions. By specifically targeting the R132H mutation through B-cell cloning, a novel rabbit monoclonal antibody, MRQ-67, was produced that can recognize mutant IDH1 R132H and does not react with the wild type protein as demonstrated by Enzyme-linked immunosorbent assay (ELISA) and Western blotting. Through immunohistochemistry, the antibody is able to highlight neoplastic cells in glioma tissue specimens, and can be used as a tool in glioma subtyping. Immunohistochemistry (IHC) detection of IDH1 mutant protein may also be used to visualize single infiltrating tumor cells in surrounding brain tissue with an otherwise normal appearance.

## 1. Introduction

Isocitrate dehydrogenase 1 (IDH1) functions as an enzyme in the Krebs (citric acid) cycle and is biologically active in the cytoplasmic and peroxisomal compartments under normal conditions [1]. Somatic mutations in the gene that encodes IDH1 have been reported to be present in some glioma subtypes in high frequencies. The majority of these particular tumors have been found to harbor heterozygous point mutations in codon 132, with a missense amino acid substitution of arginine to histidine (R132H) being observed to have highest rate of occurrence [2]. The high incidence of glioma-specific IDH1 mutations has implicated them as an early event that occurs during gliomagenesis and provides utility in distinguishing low grade astrocytomas and oligodendrogliomas, as well as secondary glioblastomas from reactive gliosis and primary glioblastomas [3]. The value of this mutant marker is further illustrated by the 2016 World Health Organization (WHO) Classification of Tumors of the Central Nervous System that newly incorporates IDH1 mutation status as a parameter for sub-classifying diffuse astrocytic and oligodendrogliomal tumors [4]. While genetic testing can be burdensome, a clinically established routine procedure like immunohistochemistry (IHC), using a specific monoclonal antibody directed against IDH1 R132H mutant protein, represents a useful tool for overcoming this diagnostic challenge.

This study describes the generation and performance of a novel rabbit monoclonal IDH1 R132H antibody (MRQ-67) by single B-cell cloning technology, a recently emerging strategy for monoclonal antibody development [5]. The capacity of MRQ-67 to identify mutant IDH1 R132H without reacting with wild type protein is demonstrated through binding specificity assays. The functional utility of the antibody to specifically detect mutant IDH1 protein in astrocytomas, oligodendrogliomas, and glioblastomas is also examined through immunohistochemical analysis.

## 2. Materials and Methods

### 2.1. Tissue Specimens

Immunohistochemical evaluation of MRQ-67 performance was assessed using formalin-fixed, paraffin-embedded (FFPE) tissue specimens, which included 18 cases of astrocytoma, 7 cases of oligodendroglioma, 7 cases of glioblastoma, 12 cases of meningioma, and 15 cases of non-neoplastic brain tissue. The FFPE tissue specimens used in this study were procured, qualified, and tested in accordance with the U.S. Food and Drug Administration (FDA) “Guidance on Informed Consent for In Vitro Diagnostic Device Studies Using Leftover Human Specimens that are Not Individually Identifiable”. This study exclusively used leftover tissue specimens that are not individually identifiable for conducting IHC testing. More specifically, these are remnants of human specimens collected for routine clinical care or analysis that would have otherwise been discarded and where the identity of the subject is not known to, or may not be readily ascertained by, any individual associated with this study.

### 2.2. Immunization

New Zealand White Rabbits were immunized with synthetic peptide CKPIIIGHHAYGD coupled to Keyhole limpet hemocyanin (KLH) corresponding the amino acids 126 to 137 of the human IDH1 containing R132H mutation. All of the housing and immunization procedures were performed by Antibodies Incorporated (Antibodies Inc., Davis, CA, USA), according to the approved protocols and guidelines of the Institutional Animal Care and Use Committee (IACUC). The project numbers were 5834 and 5835 under IACUC protocol 0298-9 “Custom Polyclonal Antibody Production in Rabbits”, approved on 1 July 2016 (PHS Assurance number A4064-01).

### 2.3. Isolation and Sorting of Rabbit B-Cells

Peripheral blood mononuclear cells (PBMCs) were isolated from the ethylenediaminetetraacetic acid (EDTA) containing peripheral blood by density-gradient centrifugation with Lympholyte-Mammal (Cedarlane, Burlington, NC, USA), as described in the manual. The isolated PBMCs (10^7^ cells) were next washed with RPMI (Life Technologies, Carlsbad, CA, USA) containing DNase I (Roche, Basel, Switzerland) and re-suspended in phosphate-buffered saline (PBS) containing 0.5% bovine serum albumin (BSA). Then, the IgG expressing B-cells were isolated using Anti-Rabbit IgG Microbeads (Miltenyi Biotech, Auburn, CA, USA).

The isolated B-cells were stained for viability, incubated with a cocktail of anti-rabbit IgG and fluorochrome-conjugated specific peptide in the dark for 30 min at 4 °C, and washed with ice-cold PBS. Finally, cells were re-suspended in PBS and subjected to Fluorescence Activated Cell Sorting (FACS) analyses. Sorting was carried out using BD Influx Cell Sorter and BD FACS DIVA software (UC Davis Medical Center, Sacramento, CA, USA). Single B-cells expressing IgG were sorted into a 96-well plate (omitting row H).

### 2.4. Cloning Antibody Variable Regions

Single-cell reverse transcription polymerase chain reaction (RT-PCR) was performed with reverse transcriptase Superscript III First-Strand Synthesis system (Life Technologies, Carlsbad, CA, USA). Next, the RT-PCR reaction mixtures were used for subsequent polymerase chain reaction (PCR) reactions to amplify variable regions of IgG heavy chain (V_H_) and light chain (V_L_). The V_H_ and V_L_ were separately cloned in pTrans-CMV-MCS expression vectors (CEVEC, Köln, Germany) containing constant region coding sequences for rabbit IgG γ and IgG κ.

### 2.5. Transfection

Primary human amniocytes CAP-T cells (CEVEC, Köln, Germany) were transiently co-transfected with vectors containing the codon sequences of heavy chain and light chain originating from the same sorted cell using NovaCHOice^®^ transfection kit (MilliporeSigma, Billerica, MA, USA), and the supernatants were harvested after seven days for evaluation by Enzyme-linked immunosorbent assay (ELISA), Western blotting, and IHC.

### 2.6. ELISA

The concentration of the IgG released by transfected cells was measured using Rabbit IgG ELISA kit (ZeptoMetrix Corp., Franklin, MA, USA). Serial dilutions of a rabbit IgG antibody (60, 30, 15, 7.5, 3.75, and 0 ng/mL), provided by the kit, were used to set up a standard curve. The specificity of the antibody was determined by immobilizing biotinylated synthetic peptides (provided by Antibodies Inc., Davis, CA, USA) KPIIIGHHAYGD (mutant) or KPIIIGRHAYGD (wild type) on a 96-well plate. The plate was coated with 2 µg/mL streptavidin (MilliporeSigma, Billerica, MA, USA) overnight at 4 °C and then the peptides were immobilized at 1 µg/mL for one hour at room temperature. After blocking with 5% skim milk for an hour, the plate was probed with the supernatant of transfected cells or a commercially available mouse monoclonal IDH1 R132H H09 antibody (Dianova, Hamburg, Germany) at different concentrations, starting from 1 µg/mL and lower and incubated for an hour at 37 °C. Next, a peroxidase-conjugated anti-rabbit IgG anibody (Jackson ImmunoResearch Lab, West Grove, PA, USA) (1:1000) was added to the plate and incubated for an hour at room temperature. The enzymatic reaction was conducted with Tetramethylbenzidine (TMB) (MilliporeSigma, Billerica, MA, USA) at room temperature and stopped by 0.25 M Sulfuric acid (MilliporeSigma, Billerica, MA, USA). The optical density was measured at 450 nm. The plate was washed four times after every step with PBS containing 0.05% Tween 20 (MilliporeSigma, Billerica, MA, USA).

### 2.7. Western Blotting

100 ng of recombinant human IDH1 R132H protein and wild type IDH1 protein (Abcam, Cambridge, MA, USA) were loaded onto 4–12% Bis-Tris mini gels (Life Technologies, Carlsbad, CA, USA) and blotted to PVDF membranes (iBlot™ Transfer Stack, PVDF, Invitrogen, Carlsbad, CA, USA). The Western blot was carried out using WesternBreeze Chromogenic kit (Invitrogen, Carlsbad, CA, USA) and the supernatant of the transfected cells (0.5 µg/mL IgG) or H09 (0.5 µg/mL antibody) were used to probe the membranes.

### 2.8. Immunohistochemistry

FFPE tissue samples were sectioned at a thickness of 4 µm and were prepared on Superfrost™ Plus microscope slides (Fisherbrand™, Pittsburgh, PA, USA). Prepared slides were stained by routine IHC on a BenchMark ULTRA automated staining instrument (Ventana Medical Systems Inc., Tucson, AZ, USA). Tissue slides were incubated for 64 min at 95 °C using an EDTA-based epitope retrieval solution, followed by a 32 min primary antibody incubation at 36 °C. Staining signal was visualized through a horseradish peroxidase (HRP)-based multimer detection system and 3-3′-Diaminobenzidine (DAB) chromogen. Counterstaining was performed by incubating tissue slides for 4 min with Hematoxylin II, followed by a 4 min incubation with bluing reagent. Stained slides were evaluated using light microscopy for target signal intensity and background signal by a qualified pathologist. Tumor cells exhibiting a strong, diffuse cytoplasmic staining pattern, as well as weaker nuclear labeling, were scored as positive for IDH1 mutant protein. The H09 antibody was used as a reference comparison during performance testing of the antisera from immunized rabbits and optimization of the selected MRQ-67 clone. The H09 clone further served in establishing the IDH1 R132H mutation status of glioma samples that were used in this study. The same automated staining conditions were used for both the MRQ-67 and H09 clones, with optimal antibody titers having been experimentally determined for each clone individually. The MRQ-67 and H09 clones were determined to perform optimally in IHC at concentrations of 2.54 µg/mL and 3.25 µg/mL, respectively.

## 3. Results

Four rabbits were immunized with the synthetic IDH1 R132H mutant peptide. Sera from immunized rabbits were tested by IHC. The PBMCs from the rabbit with the best immune response were isolated for cloning of V_H_ and V_L_, followed by co-transfection in CAP-T cells.

**Figure 1:**
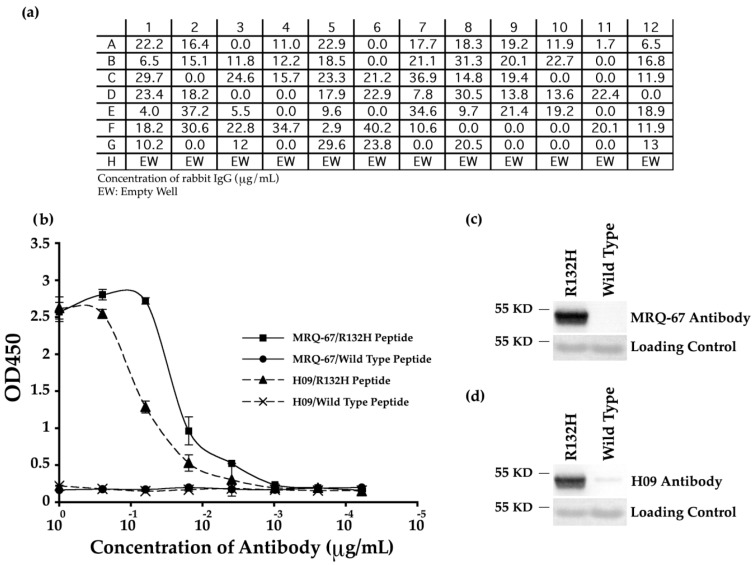
Generation and performance of rabbit monoclonal MRQ-67 antibody against IDH1 R132H and comparing its function with the H09 antibody by Enzyme-linked immunosorbent assay (ELISA) and Western blotting assays: (**a**) The concentration of antibody generated by clones was measured in µg/mL by rabbit IgG ELISA kit; (**b**) Peptide binding ELISA. MRQ-67 antibody specifically recognized only mutant IDH1 R132H peptide and not wild type IDH1 peptide. The ELISA binding assay result for the H09 antibody has been included as a control; (**c**,**d**) Analysis of MRQ-67 and H09 for detecting recombinant human IDH1 R132H and recombinant human IDH1 wild type proteins in Western blotting. MRQ-67 (**c**) and H09 (**d**) antibodies (0.5 µg/mL) were used to probe the membranes. Coomassie blue stainings of the proteins are shown as loading control.

ELISA analyses of the transfection reactions confirmed the production of rabbit IgG by 73% of the clones (Figure 1a). The positive clones were further screened for specificity of the antibodies and among them, antibody MRQ-67 generated by clone C5 was selected for further evaluations. As shown in Figure 1b, the MRQ-67 antibody specifically reacted with mutant peptide IDH1 R132H in a dose-dependent manner, but not with the wild type peptide IDH1 in ELISA assay, indicating that MRQ-67 specifically recognized IDH1 R132H. This result was consistent with the result obtained using the control antibody, H09 (Figure 1b).

The MRQ-67 antibody’s specificity was further analyzed by Western blotting and compared with the specificity of H09 antibody. As shown in Figure 1c,d, MRQ-67 and H09 both detected the recombinant IDH1 R132H protein at a predicted molecular weight of 48 kDa for human IDH1 protein. Notably, while MRQ-67 antibody did not detect any band on the blot with wild type recombinant IDH1 protein (Figure 1c), the H09 antibody showed a weak reaction with this protein (Figure 1d). Overall, this data indicates that MRQ-67 is also useful in detecting not only IDH1 R132H peptide, but also the mutant IDH1 R132H protein.

**Figure 2:**
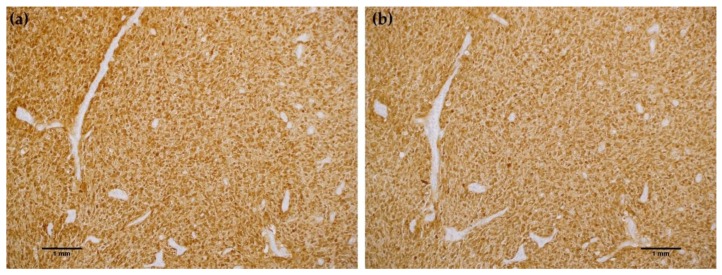
Comparison immunohistochemistry (IHC) staining results with MRQ-67 and H09 in astrocytoma: (**a**) Strong, diffuse cytoplasmic and weak nuclear labeling of tumor cells with MRQ-67 in a case of anaplastic astrocytoma (100×); (**b**) Equivalent cytoplasmic and nuclear staining of tumor cells with H09 in the same case of astrocytoma (100×).

**Figure 3:**
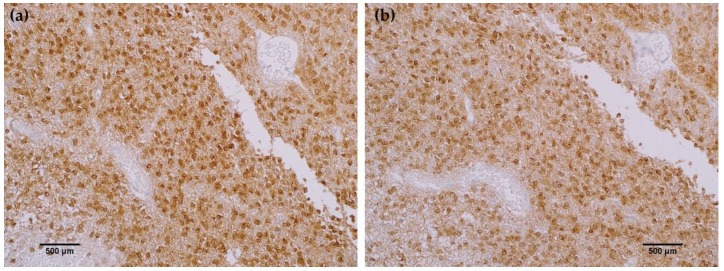
Comparison immunohistochemistry (IHC) staining results with MRQ-67 and H09 in oligodendroglioma: (**a**) Strong cytoplasmic and weak nuclear labeling of tumor cells with MRQ-67 in a case of WHO grade II oligodendroglioma (200×); (**b**) Comparable cytoplasmic and nuclear staining of tumor cells with H09 in the same case of oligodendroglioma (200×).

**Figure 4:**
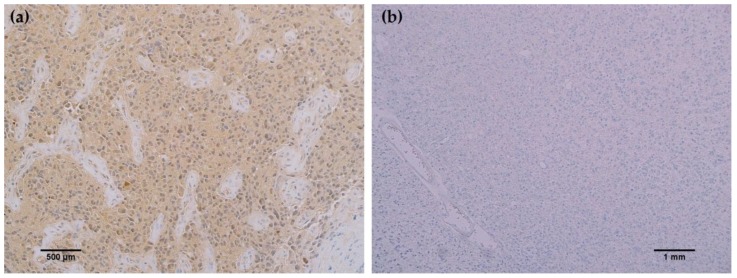
MRQ-67 immunohistochemistry (IHC) staining results in high grade glioblastoma: (**a**) Weak, diffuse cytoplasmic and nuclear labeling of tumor cells in a case of WHO grade IV glioblastoma with anaplastic astrocytoma involvement (200×); (**b**) No observed reactivity in tumor cells in another case of WHO grade IV glioblastoma (100×).

To further characterize the rabbit IDH1 R132H monoclonal antibody, the capacity of the MRQ-67 antibody to immunohistochemically identify IDH1 mutant protein in FFPE tissues was investigated. The IHC staining results summarized in Table 1 demonstrate equivalent sensitivity and specificity performance of the MRQ-67 clone in comparison to H09 for distinguishing low grade gliomas from glioblastomas, meningiomas, and benign brain samples. The MRQ-67 clone generated strong, diffuse cytoplasmic staining with weaker nuclear reactivity in 50% of the diffuse and anaplastic astrocytomas (Figure 2a) that was equivalent to performance observed with H09 (Figure 2b). Positive tumor staining in 71% of oligodendroglioma samples by MRQ-67 was primarily demonstrated by cytoplasmic reactivity (Figure 3a) and was comparable to observed results from H09 testing (Figure 3b). From the seven high grade glioblastoma cases that were tested, only one reacted positively with MRQ-67 as indicated by weakly diffuse cytoplasmic and nuclear staining (Figure 4a), while no tumor cells were labeled in the rest of the tested cases (Figure 4b).

**Figure 5:**
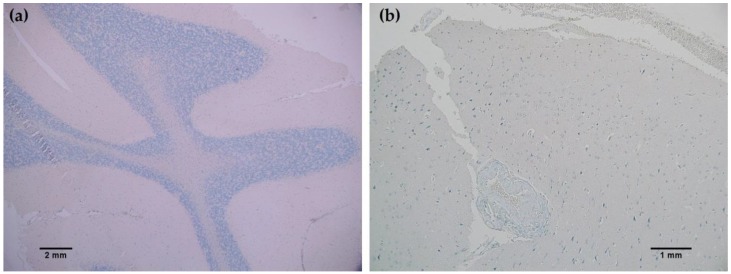
MRQ-67 immunohistochemistry (IHC) staining results in normal brain: (**a**) No observed reactivity in the normal cell types that constitute the gray and white matter in the cerebellar region of the brain (40×); (**b**) No observed reactivity in normal cerebral nervous tissue (100×).

**Figure 6:**
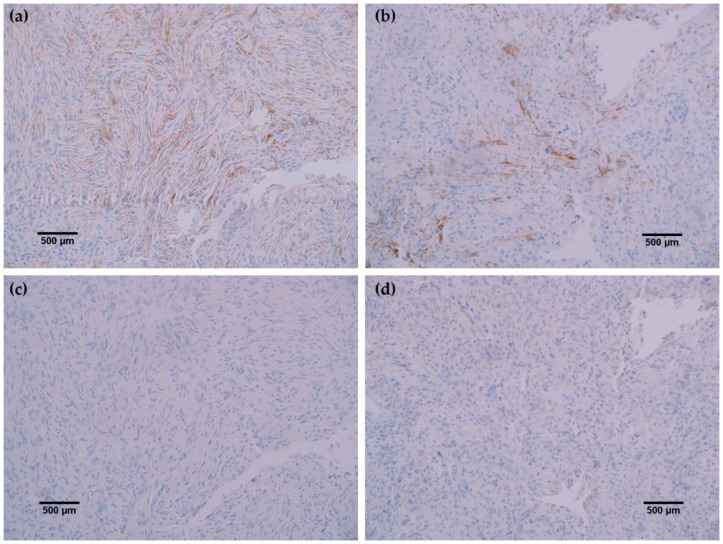
Comparison immunohistochemistry (IHC) staining results of MRQ-67 and H09 in meningioma specimens: (**a**) The H09 clone displayed positive reactivity in spindle cells of a meningioma sample (200×); (**b**) Positive labeling of fine fibrous elements by the H09 clone in another case of meningioma (200×); (**c**) The MRQ-67 clone demonstrated no cross-reactivity with spindle cells in the same meningioma case stained with the H09 clone (200×); (**d**) MRQ-67 also did not generate cross-reaction with fibrous elements as was observed with H09 staining in the same case of meningioma (200×).

**Figure 7:**
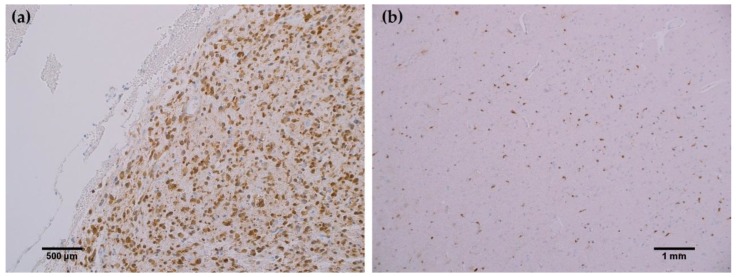
MRQ-67 immunohistochemistry (IHC) staining results in a primarily benign brain sample with a small tumor focus and some scattered tumor cells: (**a**) Cytoplasmic and weak nuclear labeling of cells within a small tumor focus of a benign brain sample (200×); (**b**) Scattered reactivity in single infiltrative tumor cells of the same benign brain case that have dispersed from the focal tumor site (100×).

In all cancerous and benign brain samples tested, the MRQ-67 antibody did not react with endothelial cells, lymphocytic cells, or normal glial cells. The 15 cases of benign brain (Figure 5a,b) and 12 cases of meningioma (Figure 6c,d) that were assessed displayed no observable cross-reactivity, with only two cases (16%) of meningioma demonstrating equivocal background staining signal when tested using MRQ-67. However, meningioma samples stained with the H09 clone generated nonspecific background signal in fibrillar and spindle cell components in 11 out of 12 cases (92%), with 4 cases (33%) in particular developing notably strong background staining (Figure 6a,b). One particular case of benign brain was observed to have a small focus of tumor cells that was identified by anti-IDH1 R132H (Figure 7a). Importantly, the surrounding distal nervous tissue was identified to consist of single infiltrative tumor cells that exhibited strong positive reactivity (Figure 7b).

## 4. Discussion

In the specific targeting of the IDH1 R132H mutation through B-cell cloning, a novel rabbit monoclonal antibody was produced that promises to be a useful tool for overcoming the diagnostic challenge of differentiating between different subtypes of glioma. The single B-cell cloning technology used to generate MRQ-67 is an attractive method for developing high quality monoclonal antibodies that has several advantages over previously established techniques. This technology is more efficient than cell fusion in hybridoma technique [6], and unlike display methods (e.g., phage display [7] and yeast surface display [8]), both heavy and light chains originate from a single sorted cell. This allows for the natural cognate pairing of the heavy and light chains to be preserved during synthesis and maturation of the antibody [5]. Moreover, culturing of isolated B-cells is not required, which removes a potential source of technical complications from the process.

In ELISA assays and Western blotting analyses, the MRQ-67 rabbit monoclonal antibody reacted with mutant IDH1 R132H, but not with wild type IDH1. This data indicates that MRQ-67 is capable of specifically detecting not only IDH1 R132H peptide, but also the mutant IDH1 R132H protein without cross-reacting with wild type IDH1. The specificity of MRQ-67 to the R132H mutation had not been tested against other less frequent mutations of IDH1 at the time of this report.

Identification of IDH1 mutation status by IHC presents a useful tool for many diagnostic institutions where genetic testing can be burdensome and may be inaccurate, especially regarding tissue samples with low tumor cell content. Detection by IHC provides the opportunity to visualize even single infiltrating tumor cells in otherwise normal appearing brain tissue. The IDH1 status of FFPE tissue samples used in this study had not been determined by sequencing at the time of this report. IDH1 mutation status of these tissues was therefore established through the use of the commercially available mouse monoclonal IDH1 R132H (H09) antibody.

Rabbit monoclonal anti-IDH1 R132H (MRQ-67) labeled each of the same cases as H09, indicating comparable ability to detect IDH1 mutant protein by IHC. However, the H09 clone generated nonspecific background signals in the fibrillar and spindle cell components in nearly all meningioma cases tested, with a few cases in particular developing notably strong background staining. The observed cross-reaction with fibrous elements in meningioma cases has been previously identified as nonspecific binding by anti-IDH1 R132H to extracellular matrix protein or a subtype of collagen fiber [9]. Staining with the MRQ-67 clone demonstrated only two instances of weak, nonspecific background signal in meningioma samples, indicating a particular advantage in specificity compared to the H09 clone. Since no immunoreaction was observed in meningioma tumor cells or normal brain samples, all tumor cells that stained are considered to be IDH1 mutant-positive, including the single infiltrating cells that dispersed from the primary tumor focus as seen in Figure 7.

The presence of IDH1 mutations has been indicated to be much more frequent in secondary glioblastomas compared to primary glioblastomas [10]. Clinical information regarding the progression of the single case of glioblastoma in this study with identified mutant IDH1 staining signal was not available, but histopathological evidence of lower grade glioma involvement was observed. Further, population-based data indicates a considerably greater incidence rate of primary glioblastoma compared to that of secondary glioblastoma [11]. These observations, together with H09 comparison staining data in glioblastoma samples, suggest that MRQ-67 functions as intended for IHC applications.

Overall, the results from ELISA assays, Western blotting, and IHC analyses support the proposed utility of the novel rabbit monoclonal MRQ-67 antibody in the identification of mutated human IDH1 protein. IDH1 (MRQ-67) rabbit monoclonal antibody is a specific marker for immunohistochemical detection of IDH1 mutant protein in glioma subtypes and may have value as a tool in distinguishing between diffuse astrocytomas and oligodendrogliomas from secondary glioblastoma.

## Figures and Tables

**Table 1:** IDH1 R132H immunohistochemistry (IHC) staining data. Summary of IHC staining results comparing the number of cases stained positive out of the total number of cases tested with the MRQ-67 and H09 clones.

Tissue	Cases Stained (MRQ-67)	Cases Stained (H09)
Astrocytoma	9/18 (50%)	9/18 (50%)
Oligodendroglioma	5/7 (71%)	5/7 (71%)
Gliobastoma	1/7 (14%)	1/7 (14%)
Meningioma	0/12	0/12
Non-neoplastic Brain	0/15	0/15
